# A Delayed Response in the Area‐Concentrated Search Can Improve Foraging Success

**DOI:** 10.1002/ece3.71173

**Published:** 2025-03-23

**Authors:** Thotsapol Chaianunporn, Thomas Hovestadt

**Affiliations:** ^1^ Department of Environmental Science, Faculty of Science Khon Kaen University Khon Kaen Thailand; ^2^ Department of Animal Ecology and Tropical Biology, Theoretical Evolutionary Ecology Group University of Würzburg, Biocenter Würzburg Germany

**Keywords:** area‐concentrated search, delayed‐response, individual‐based model, spatial memory, survey movement

## Abstract

Area‐concentrated search (ACS) is a simple movement rule implying that an animal searches for resources using a “state‐dependent correlated random walk.” Accordingly, a forager increases its searching intensity by reducing the directionality of movement (“intensive search mode” or ISM) when it detects a resource item, but if it searches unsuccessfully for a while, it returns to a more straight‐line movement to search for new resource locations elsewhere (“extensive search mode” or ESM). We propose a modified ACS, called delayed‐response ACS (dACS), which is more efficient in resource collection than standard ACS. Instead of immediately switching from ESM to ISM when encountering a resource, as is done in standard ACS, an individual foraging in the dACS mode delays this switch by x steps so that it continues moving in a straight line for a while before switching to ISM. Our results showed that an individual with a suitable delay parameter x for the dACS achieved substantially higher foraging success than an individual with standard ACS (x=0). Optimal foraging success occurred when x was approximately similar to the patch radius r. This is because, with dACS, an individual can penetrate deeper into and stay longer within a cluster, ultimately increasing the number of resources collected. Modifying the half‐saturation constant h also affected the success of foraging, but the effects depended on resource density and cluster size. Generally, h modulated the optimal x value only slightly. dACS can be interpreted as a survey movement within a resource cluster before switching from ESM to ISM. The dACS rule does not rely on complex spatial memory but only on memorizing how long ago resources were found or not. It may thus occur in a wide range of taxa, from organisms without a central nervous system to animals with complex brain systems.

## Graphical abstract

We propose a modified area‐concentrated search (ACS), called delayed‐response ACS (dACS), which is more efficient in resource collection than standard ACS. Instead of immediately switching from “extensive search mode” ESM to “intensive search mode” (ISM) when encountering a resource, as is done in standard ACS, an individual foraging in the dACS mode delays this switch by “*x*” steps so it continues moving in a straight line for a while before switching to ISM. Our results show that an individual with a suitable delay parameter “*x*” for the dACS achieves substantially higher foraging success than an individual with standard ACS.

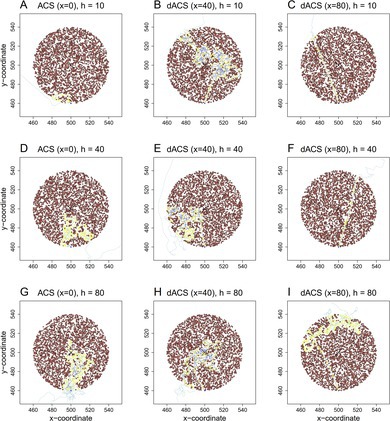

## 1 Introduction

Seeking essential resources such as food, mates, or nesting sites drives much of the movement observed in mobile animals. To be successful, such searching behavior requires continuous updating and processing of information. Some studies thus proposed a conceptual framework for movement ecology that considers the interplay among mechanistic components of movement: the internal state, motion, the navigation capacities of individuals, and the external factors affecting movement (e.g., Getz and Saltz 2008; Nathan et al. 2008). The core concept here and in other related ideas is that individuals typically have a reason or motivation to move and that they gather and process information to guide their movements. Several authors have already developed movement models that incorporate some or all of these components of movement (e.g., Avgar et al. 2013; Barton et al. 2009; Bartoń and Hovestadt 2013; Bartumeus and Catalan 2009; Benhamou 1992; Chaianunporn and Hovestadt 2022; Fagan et al. 2017, 2013; Fronhofer et al. 2013; Fryxell et al. 2008; McNamara et al. 2006; Olsson and Brown 2010; Reynolds 2012; Van Moorter et al. 2009).

Area‐concentrated search (ACS; also known as “area‐restricted search” or ARS) is a simple movement rule that can be beneficially applied whenever critical resources are heterogeneously distributed. It implies that an animal searches for resources using a “state‐dependent correlated random walk.” Accordingly, a forager increases searching intensity by reducing the directionality of movement when it detects a resource item; this is referred to as the “intensive search mode” or ISM. However, if it searches unsuccessfully for a while, it returns to a more straight‐line movement to search for new resource locations, we call this the “extensive search mode” or ESM (Benhamou 1992; Bartoń and Hovestadt 2013). Changes in searching behavior in ACS may be influenced by factors such as habitat features (Turchin 1991), diffuse cues like the scent of prey (e.g., Nolting et al. 2015), or an individual's internal state (e.g., hunger level, past foraging success, or recent prey encounters). This search rule has been implemented in many ecological studies (such as Bartoń and Hovestadt 2013; Benhamou 1992, 2004; Chaianunporn and Hovestadt 2022; Kareiva and Odell 1987; Railsback and Grimm 2012; Turchin 1991). ACS movement strategies are used to explain movement in various species (reviewed in Dorfman et al. 2022) such as amoebas (Van Haastert and Bosgraaf 2009), ladybird beetles (Nakamuta 1985), mallards (Klaassen et al. 2006), and wandering albatrosses (Weimerskirch et al. 2007).

The ACS movement strategy is quite simple and requires limited cognitive abilities of individuals only. Apart from general navigational competence like keeping a certain direction, the only requirement is some sort of memory or perception for things such as the time past the last encounter with a resource. However, more advanced individual spatial memory enables animals to navigate their environment more effectively, locate and exploit resources more efficiently, and avoid wasted efforts in previously foraged or depleted areas (Benhamou 1994; Boyer and Walsh 2010; Bracis et al. 2015; Fronhofer et al. 2013; Nabe‐Nielsen et al. 2013; Vincenot et al. 2015).

Chaianunporn and Hovestadt (2022) found that individuals that directly switch from extensive to intensive searching mode upon encountering a resource item may quickly “drift” out of a resource cluster again, leading to a brief residence time inside the cluster and thus reduced resource exploitation. For this reason, we suggest a simple modification of the ACS rules that may improve foraging efficiency compared to a standard ACS that does not include this modification. More specifically, we propose the following improvement to the rule: delayed‐response area‐concentrated search (dACS). Instead of immediately switching from intensive search (ISM) to extensive search mode (ESM) upon encountering a resource, an individual delays this switching and continues on its straight‐line movement for a while before the switching to ISM. This strategy modification can impact the duration spent within a resource cluster, the distance animals penetrate into a cluster, and the number of resources harvested. We tested the effects of the delayed‐response ACS across different parameter ranges, including resource cluster size and density and the half‐saturation constant of the movement; the latter affects how quickly a forager returns to more straight‐line movement (ESM) after foraging unsuccessfully for a while.

## 2 Methods

We simulated the movement paths of an individual in a continuous landscape with a heterogeneous resource distribution. Because we are interested in the resource exploitation of single clusters only, we consider just the situation of individuals approaching and ultimately leaving a single resource cluster. Consequently, in our simulation landscapes, resource items are distributed in a single cluster and the individual searches for and collects resources using an area‐concentrated search strategy with varying degrees of delayed switching from extensive to intensive search mode.

### 2.1 Spatial Distribution of Resources and Scenarios

For the simulations we created infinite landscapes with a single resource cluster. The cluster size and resource density varied among different scenarios. We utilized the Matérn Cluster Point Process using R version 3.5.3 and the “spatstat” library version 1.58–2 to create resource clusters as a continuous spatial point pattern (Baddeley and Turner 2005). To understand the effects of the two landscape parameters, patch radius *r* and resource density *u* on foraging success (see below), we created landscapes with either the cluster radius fixed at r=40 and resource density u set to 0.04, 0.16, or 0.64 resource items per unit area respectively, or with resource density fixed at u=0.16 and cluster radius varied from 20 to 40 and 80 (measured in step length). The expected number of active resource items per cluster ($\bar{R}$) is consequently calculated as $\bar{R}=g_{i}^{2}\pi \times u$. A summary of all model and simulation parameters and their values can be found in Table 1.

**TABLE 1:** Definition and ranges of parameters values used.

Symbol	Description	Values
r	Radius of cluster [distance] (scenarios with fixed resource density (u) at 0.16 resources per unit area)	20, 40, 80
u	Resource density [resource items/area] (scenarios with fixed cluster radius (r) at 40 units)	0.04, 0.16, 0.64
$d_{\min}$	Minimum value for correlation of turning angles of consecutive steps	0.01
$d_{\max}$	Maximum value for correlation of turning angles of consecutive steps ($d_{\max}=1$) results in straight line movement if $\Delta_{S\left(x\right)}\to \infty$	1
α	Shape parameter	3
h	Half‐saturation constant	10, 20, 40, 80
p	Step length of movement [distance]	1
c	Perception radius [distance]	1
x	Delay parameter	0, 2, 4, 6, …100 (maximum value of x=200 only in scenarios with r=80)

### 2.2 Movement Rule and Foraging

As a movement rule, we implemented an area‐concentrated search where an individual switches between two movement modes: intensive search mode (ISM) characterized by low directionality of movement, and extensive search mode (ESM) characterized by high directional persistence. This is referred to as the standard ACS rule. At each time step, an individual moved one step with a constant step length (p=1 spatial unit). After moving, an individual immediately harvested all resource items within its perception radius (c=1 spatial unit). The removed resource items were not replaced after harvesting, so that resource density in the cluster might degrade over time. We assumed that an individual moved straighter, the longer the searching time ($\Delta_{S}$), that is, the time passed since the last encounter with a food item (Bartoń and Hovestadt 2013; Benhamou 1992). If an individual found at least one resource item, the searching time was set to 0 ($\Delta_{S}=0$) and the ISM was initiated (see equation 1). Whenever an individual did not find a resource item, the value of $\Delta_{S}$ for the individual was increased to $\Delta_{S}+1$. The turning angle for the next movement step was then determined by sampling a random value from a wrapped circular normal distribution (Jammalamadaka and SenGupta 2001) with mean of zero and a standard deviation $d_{t}\left(\Delta_{S}\right)$ creating from the “rwrappednormal” function in R‐package “circular” (Version 0.5‐0; Agostinelli and Lund 2023) calculated as:

(1) $$d_{t}\left(\Delta_{S}\right)=d_{\min}+\left(d_{\max}-d_{\min}\right)\left(1-\frac{\Delta_{S}^{\alpha }}{\left(\Delta_{S}^{\alpha }+h^{\alpha }\right)}\right)$$

The minimum value of $d_{t}$ was equal to $d_{\min}=0.01$ (nearly straight‐line movement—ESM) when $\Delta_{S}\gg h$ (the half‐saturation constant), and its maximum value was equal to $d_{\max}=1$ when $\Delta_{S}=0$, that is, in case the individual just found a food item. In the latter case, the movement became highly uncorrelated (ISM), and the individual performed an area‐concentrated search (see Chaianunporn and Hovestadt 2022 for more details). The shape parameter was fixed at α=3 throughout all simulations. However, between scenarios, we varied the half‐saturation constant (*h*) from 10 to 80 (see below). The half‐saturation constant regulates the shift from ISM to ESM: A higher half‐saturation constant means that a higher $\Delta_{S}$ value is required to switch back to the extrinsic movement mode.

**FIGURE 1:**
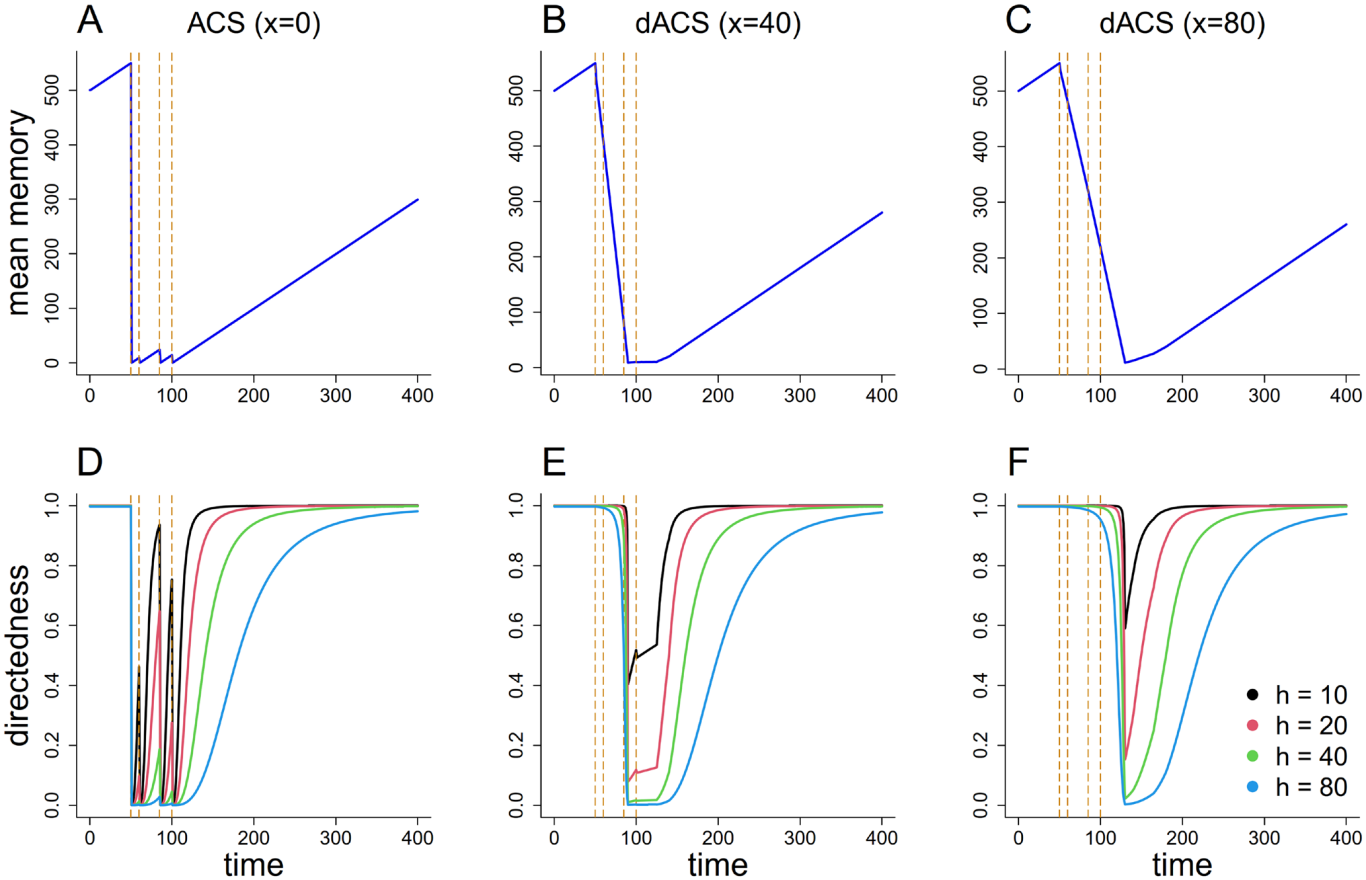
Examples of change in mean memory (top panel) and directedness of movement (bottom panel) with different values of half‐saturation constant h and magnitude of delay x in ACS and dACS when an individual acquires a resource item at the time 50, 60, 85, and 100 (dashed lines): (A and D) No delayed or standard ACS (x=0), (B and E) dACS with x=40 and (C and F) dACS with x=80.

**FIGURE 2:**
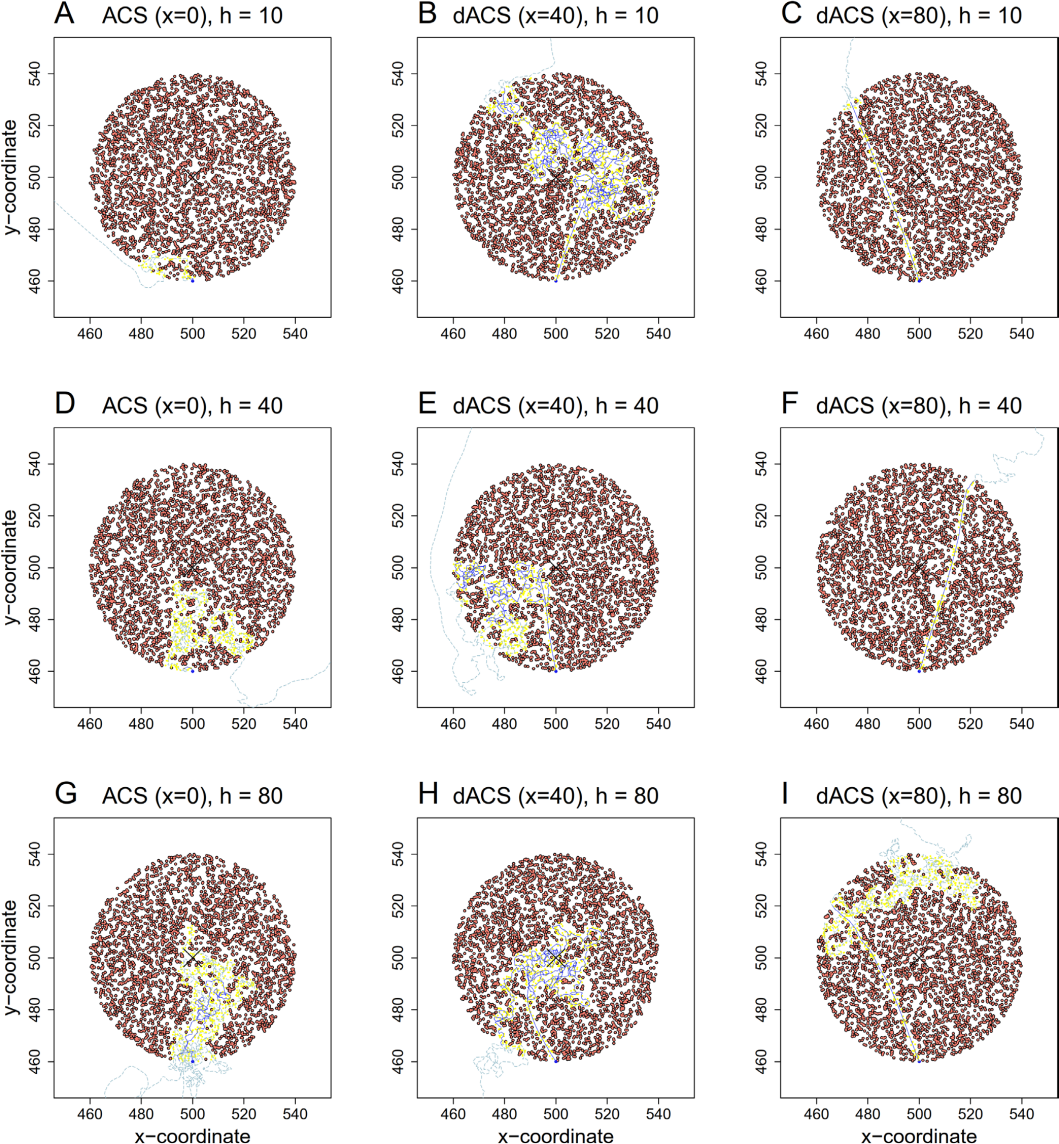
Examples of movement paths with different values of the half‐saturation constant (h) and the magnitude of the delay (x) (the cluster radius and resource density are fixed at r=40 and u=0.64, respectively). Orange dots indicate the positions of resource items, and a black cross indicates the cluster center. A blue line shows the movement path during the first cluster visitation, and a light‐blue dashed line shows the movement path after the first cluster visitation. Resource items harvested by an individual were removed from the cluster (yellow dots): (A) No delayed or standard ACS (x=0) and h=10, (B) dACS with x=40 and h=10, (C) dACS with x=80 and h=10, (D) no delayed or standard ACS (x=0) and h=40, (E) dACS with x=40 and h=40, (F) dACS with x=80 and h=40, (G) no delayed or standard ACS (*x*=0) and *h*=80, (H) dACS with *x*=40 and *h*=80, (I) dACS with *x*=80 and *h*=80.

With the standard rule for the ACS just described, a moving animal switched immediately to ISM the moment it encountered a resource item as $\Delta_{S}$ is set to zero at that moment. To allow for a more delayed transition from extensive to intensive searching we implemented a modified version of this rule by introducing a prolonged (or cumulative) “memory vector” of searching times over the previous x time steps that affects the calculation of $\Delta_{S}$. More specifically, we replace $\Delta_{S}$ in equation (1) by the quantity

(2) $$\Delta_{S\left(x\right)}=\frac{\sum_{\delta =0}^{\delta =x}\Delta_{\delta }}{x+1}$$

with x+1 as the total length of the memory vector (including the very last value memorized just now) and $\Delta_{\delta }$ as the search time memorized δ time (viz. movement) steps before the last. $\Delta_{S\left(x\right)}$ is thus simply the mean search time memorized in the last x+1 movement steps. The standard ACS was consequently recovered by setting x=0. For practical reasons, we only used even values for x in our simulations, that is, we incremented x in steps of 2. Calculating search time as average over several past movement steps led to a delayed switching to ISM after finding first resources, the more so, the larger the value of x (See Figure 1 for example). In the following, we named this modified version of the ACS “delayed‐response area‐concentrated search,” abbreviated to “dACS.” Examples of movement paths with some parameter combinations (x=0,40, and 80, h=10,20,40, and 80, r=40 and u=0.64) are presented in Figure 2.

### 2.3 Simulations and Analysis

For each combination of movement parameters (x,h; see below) we simulated movement in landscape with the following combinations of two landscape parameters: (1) resource density ($u\in \left\{0.04,0.16,0.64\;\right\}$ with cluster radius fixed at r=40), or (2) cluster size ($r\in \left\{20,40,80\right\}$) with resource density fixed at u=0.16, that is, 3+4=7 different landscape setting in total. To create summary statistics, we replicated simulations of 40 individuals independently for each of these parameter combinations.

In each simulation run, a single individual was released at the southern edge of the resource cluster. The movement of animals was characterized by any combination of the two movement parameters $x\in \left\{0,2,4,\ldots 100\right\}$ (except in r=80 where the maximum value of x is 200) and $h\in \left\{10,20,40,80\right\}$. The results presented later are consequently based on a total of 65,120 individual simulation runs (N=40×7×51×4=57120) plus 8000 (N=40×50×4) extra simulations for the scenario r=80. At the beginning of each run, the memory vector $\Delta_{\delta =0}\ldots \Delta_{\delta =x}$ was initialized with the vector 500+x/2,…500,…500−x/2 so that $\Delta_{S\left(x\right)}=500$, ensuring that individuals exhibited nearly straight‐line movement (thus assuming that the individual had traveled for considerable time before reaching the resource cluster). The initial direction of the individual was randomly selected from a uniform distribution between 1/4π and 3/4π. At each time step, individuals moved and searched for resources as previously described. For each individual, we performed a simulation of 1000 movement steps. Individuals that never entered the resource cluster were removed from the data set and the simulation run was repeated.

We compared the efficiency of dACS in dependence of x and h using three criteria: (1) foraging success—the number of resources collected during the whole simulated run (t=1000 movement steps), (2) first cluster visitation duration—the duration of the first excursion into the resource cluster, defined as the time between when an individual entered the cluster area (even without foraging success) and when it left the area for the first time, and (3) center approximation—the shortest distance to the patch center reached during the first excursion into the resource cluster. We tested interaction effects on these metrics between the delay in dACS (value of x) and resource density u (with a fixed resource cluster size), resource cluster size r (with a fixed resource density), and the half saturation constant h. For graphical presentation, the mean value of the forty replicate simulation runs was calculated and shown in figures using a cubic smoothing spline (function “smooth.spline” in R version 4.1.0). All simulations were carried out in R version 4.1.0.

## 3 Results

In this study, we investigated the effects of a simple “memory‐based” delay x in an area‐concentrated search's transition from ESM to ISM on foraging success, patch visitation time, and approximation to patch center. In particular, we explored how the optimal value of x depends on a second behavioral parameter that controls the back‐transition from ISM to ESM (h) as well as on resource density and patch size.

### 3.1 Scenarios With Fixed Cluster Size and Varied Resource Density

**FIGURE 3:**
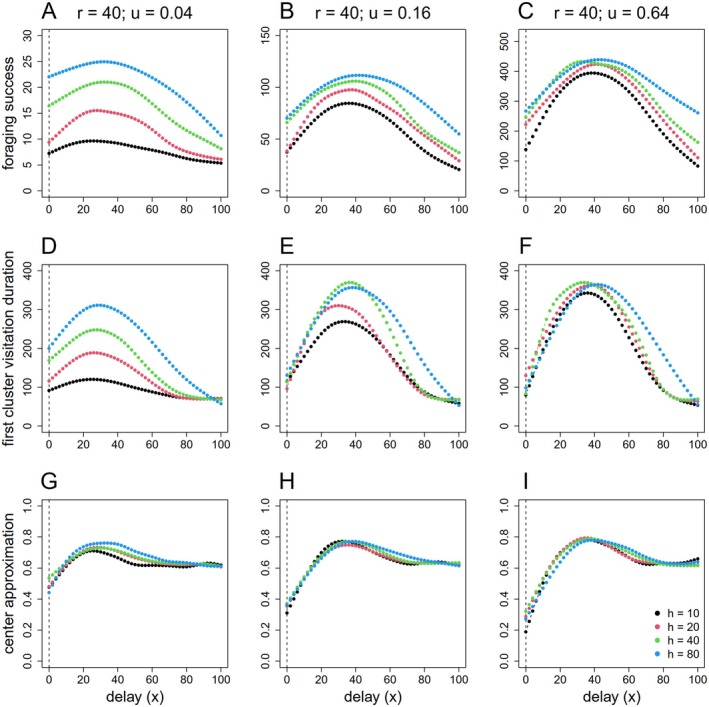
Effects of half‐saturation constant h and magnitude of delay x in dACS on foraging success (top panel), first cluster visitation duration (middle panel), and center approximation (bottom panel) in scenario with fixed cluster size (r=40) and varied resource density: (A, D, G) u=0.04, (B, E, H) u=0.16, and (C, F, I) u=0.64. The dashed lines mark the results from the standard ACS with x=0. Note that the scale of foraging success and first cluster visitation (*y*‐axis) might be varied among columns.

Across all metrics, that is, foraging success, first cluster visitation duration, and center approximation, a unimodal dependence on delay parameter x emerged (Figure 3). The dACS with a memory component (delay) showed optimal performance when the delay lasted for approximately 30–50 movement steps, with some adjustments depending on the resource density and the half‐saturation constant value (Figure 3A–C). Different half‐saturation constant values influenced the peak's height and position. Higher half‐saturation constant generally led to higher foraging success, with the peak value for x moving to slightly larger values. The sensitivity to changes in x decreased as the value of h increased. High resource density obviously enhanced foraging success, but peak positions became nearly independent of parameter h; success at peak values in fact increased approximately proportional to the increase in resource density u. Expressed differently—compared to the standard ACS (x=0)—the proportional increase in foraging success at peak values of x was larger at higher resource density and more pronounced for small values of h. Interestingly, at low values of x duration of first visits was very similar across different values of h at intermediate and high resource densities (Figure 3E,F), but foraging success (measured over the entire simulation time) was nonetheless higher with larger values of h. This can only be explained by a difference in the likelihood to return to the patch after the first visit, depending on h.

### 3.2 Scenarios With Varied Cluster Size and Fixed Resource Density

**FIGURE 4:**
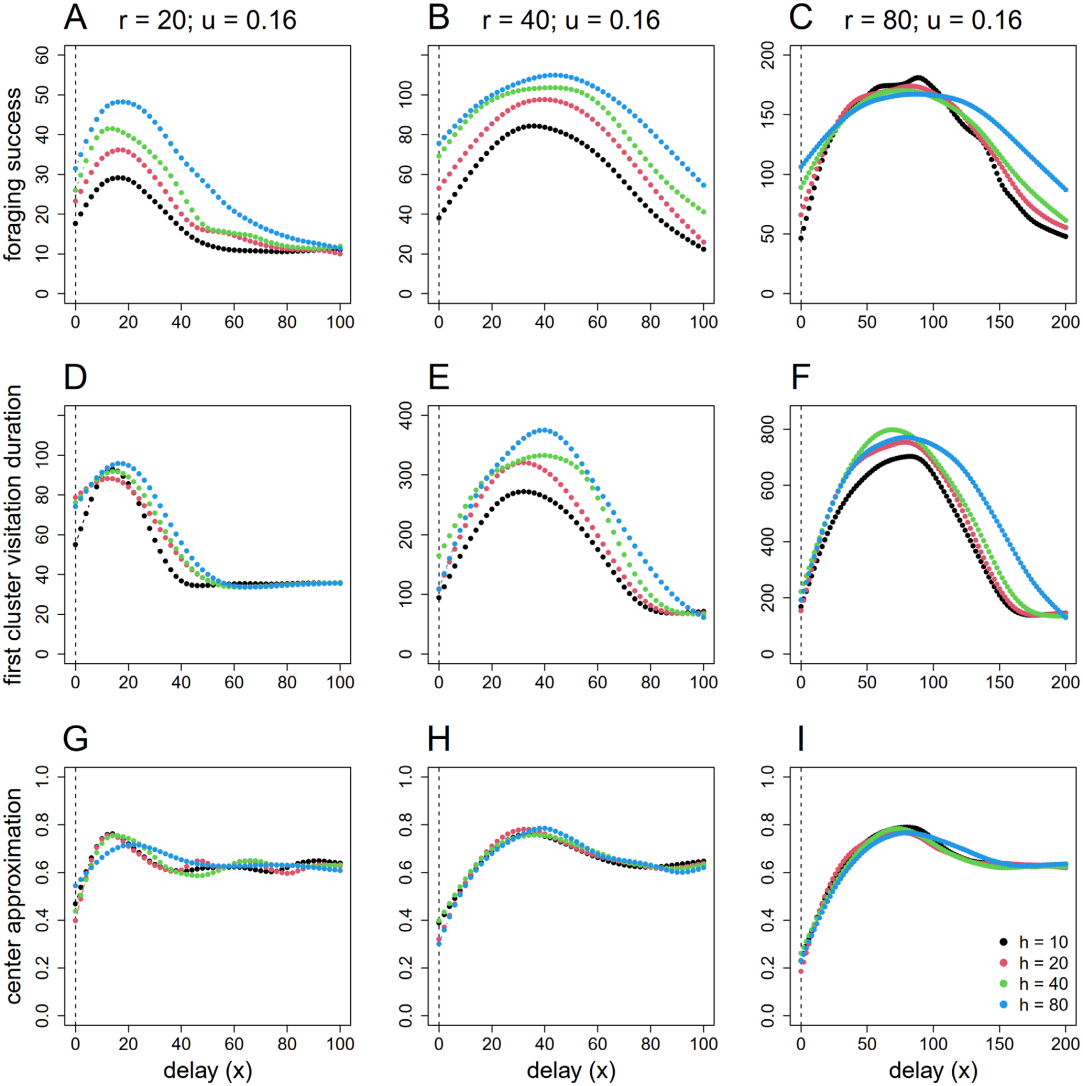
Effects of half‐saturation constant h and magnitude of delay x in dACS on foraging success (top panel), first cluster visitation duration (middle panel), and center approximation (bottom panel) in scenario with fixed resource density (u=0.16) and varied cluster size: (A, D, G) r=20, (B, E, H) r=40, and (C, F, I) r=80. The dashed lines mark the results from the standard ACS with x = 0 scenarios. Note that the scale of foraging success, first cluster visitation (*y*‐axis) and delay (*x*‐axis) might be varied among scenarios.

In the scenarios with variable cluster size r and fixed resource density u, the size of the resource cluster greatly influenced the effect of dACS on foraging success: foraging success peaked at values where x≈r (as a peak is not clearly visible for r=80 when x=100, we increased tested values up to x=200) (Figure 4). Consequently, an individual benefited from a lower delay value with a sharp peak and decline in smaller clusters while foraging in larger clusters benefited from longer delays in the switching to intensive searching. This was held for all metrics, that is, foraging success, first cluster visitation duration and center approximation, but the effects were proportionally larger for the duration of the first visit than for foraging success. Larger cluster radius generally resulted in higher foraging success and longer duration of the first visit. Center approximations were mainly dependent on the delay x (up to x≈r) but little affected by parameter h. The effects of the half‐saturation constant h on foraging success and visitation time appeared to be weaker in the large cluster scenarios (r=80) with an interesting interaction effect between h and x emerging.

## 4 Discussion

The area‐concentrated search (ACS) has been reported in a number of taxa, including protists (Latty and Beekman 2009), nematodes (Hills et al. 2004), snails (Cloyed and Dell 2019), insects (Ferran et al. 1994; Nakamuta 1985), birds (Nolet and Mooij 2002; Weimerskirch et al. 2007), mammals (Fernandez‐Betelu et al. 2023; Kays et al. 2023), and even humans (Pacheco‐Cobos et al. 2019). Several previous studies have suggested improvements to ACS (reviewed in Dorfman et al. 2022). In this study, we implemented a simple modification of ACS movement, specifically a memory based delayed‐response in the ACS (delayed‐response ACS or dACS). On the one hand, we found that even though the rule was as simple as “do not immediately switch from extensive to intensive search mode, but wait for x steps,” individuals using dACS tended to be substantially more successful than those using regular ACS with a more or less clearly defined optimum value of x. In addition, a strategy involving a slower switch from intensive to extensive search (i.e., a higher half‐saturation constant h) also tended to be more successful at resource harvesting, in particular in small patches or patches with low resource density.

Our work highlights the importance of adjusting the parameters x and h based on resource density and cluster size to maximize foraging success. In a landscape with clumped resource distribution, an individual with dACS should perform substantially better than an individual with normal ACS. Because an individual with ACS switches from ESM to ISM immediately, it typically harvests resources at the edge of the cluster and may quickly drift out of the cluster again. Sometimes it can return back to the cluster creating a “foray loop” (Chaianunporn and Hovestadt 2022), but often enough, in particular once it switches back to ESM (depending on h), it permanently moves away from the patch. In contrast, an individual with dACS that maintains its ESM and penetrates deeper into the resource cluster has a greater chance for harvesting resources from inside the cluster and staying longer in the patch (examples in Figure 2); we showed that this works best if x corresponds with the patch radius r. If x>r individuals tend to move towards the opposite edge of the resource cluster, likely leaving it as soon as x>2r (Figure 3 and Figure 4). Neither resource density nor the half‐saturation constant h show relevant interactive effects with the optimum value of x (Figure 3).

Considering the role of the half‐saturation parameter h, we find that larger h values result in larger foraging at peak x, but the advantage of larger h mostly shows when patches are small, or resource density is low. In addition, with larger h values foraging success is more robust against changes in x. Consequently, choosing a larger h may be a strategy to reduce variance in foraging success if individuals cannot know the dimension of resource clusters or resource density.

Comparing the proportional variance along x and between different h values for the foraging success and the duration of the first visits in Figures 3 and 4, we recognize that the increased foraging success associated with higher h values can only partially be attributed to the prolongation of the duration of the first visits, especially at low x values. Individuals with a higher h spend more time within the resource cluster because a higher h causes a more delayed back‐transition from ISM to ESM; this effect was previously reported by Bartoń and Hovestadt (2013). Yet a larger h value also increases the probability that an individual returns again to the patch after leaving it for the first time. Both effects contribute to the foraging success that was calculated over the whole simulation time in our experiments.

The latter effect is more prominent in scenarios with low resource density (Figure 3), as—due to a low resource finding rate—an individual with a lower h may “prematurely” switch to extensive search and move away from the resource cluster. Since this study assumes a single resource cluster, moving away from it carries the risk of not returning to the cluster within the simulation time. However, in a landscape where resource concentrations are abundant and resource densities vary, a lower h value might be beneficial overall, as a lower h value can steer animals away earlier from non‐profitable, possibly exhausted resource clusters. Changing parameter h may consequently be a behavioral mechanism that allows to control the “giving‐up time” frequently addressed in studies on optimal foraging in patchy habitats (Krebs et al. 1974; Ydenberg 1984).

In this study, we varied only the half‐saturation constant and the delay parameter for the movement strategy. Bartoń and Hovestadt (2013) reported that the shape parameter (α) generates similar effects to varying the half‐saturation constant. Furthermore, the half‐saturation constant and the shape parameter both appear to interact with different types of resource distribution in landscapes (homogeneous or heterogeneous, and fine or coarse‐grained) and the number of resource clusters. However, we believe that our finding—that dACS can improve foraging success over normal ACS—should hold true across various parameter spaces, except in some cases where resources are distributed in a fine‐grained pattern and the landscape is homogeneous. In such landscapes, the success rate of dACS and ACS may not differ significantly.

In our study we implement the delay in the dACS in a particular way. However, technically it could have been done in some alternative ways, like switching from ESM to ISM exactly after x time viz. movement steps. This should also avoid the effect parameter h—that primarily affects the back‐transition from ISM to ESM—also has on the ESM‐ISM transition (Figure 1). The impact of the delayed response sets in earlier and more gradually than with lower h values; this is a consequence of the mathematical equation given in equation (1). More importantly, individuals may not necessarily base the delay in the ESM‐ISM transition on the encounters with resource items, that is, foraging success, but also on more indirect cues signaling profitable foraging areas (e.g., Nolting et al. 2015). Such cues can be general habitat attributes, odor plumes, or even marks of previous foraging activity by conspecific. Yet any such modification in the decision rule should, in our opinion, not affect the principal findings we report here as they all would have the consequence that individuals penetrate deeper into suitable resource patches before shifting to ISM.

Our dACS rule requires only a simple cognitive system that memorizes the time since last finding food. Consequently, it can presumably be realized even in organisms without a central nervous system or with just simple brains lacking the ability to form complex spatial memories or navigational maps, as assumed in several previous studies suggesting optimal foraging rules for heterogeneous landscapes (e.g., Benhamou 1994; Boyer and Walsh 2010; Fronhofer et al. 2013; Getz and Saltz 2008; Nabe‐Nielsen et al. 2013; Vincenot et al. 2015). The delayed‐response movement we propose here can be interpreted as a survey movement within a resource cluster, during which an individual acquires information about the resource cluster and its resource distribution (Déjean et al. 1993; Eifler et al. 2012; Hill et al. 2003). During this survey process, the individual may continue extensive search movement until it has gathered enough information about the resource distribution, after which it begins intensive search movement.

Although a simple memory system is sufficient to improve search efficiency compared to standard ACS, our results also suggested that individuals might perform even better if they were capable of estimating resource cluster size and resource density to select the optimal delay x and optimal reversal to extensive search h; the former should be sensitive to cluster size and the latter rather to resource density. Such ability certainly requires more complex cognitive abilities than the unconditional rule we investigated here. Previous studies have reported that various animals, even insects, possess the ability to estimate resource patch size, which influences their foraging, oviposition, patch selection, and searching behaviors (Campbell and Runnion 2003; Eveleigh and Chant 1982; Hemptinne et al. 1996; Hill et al. 2003; Mallon and Franks 2000; Mugford et al. 2001; Szentesi 2003; Tortorici and Bell 1988; Visscher 2007). Thus, it can be assumed that, in contrast to this study, animals might even perform adaptive dACS with varying values of x and h according to cluster size and resource density. With this strategy, animals would be highly successful in harvesting resources in a patchy landscape.

## 5 Conclusions

In this study, we modified the standard ACS rule to a delayed‐response ACS rule (dACS), a strategy that does not rely on spatial memory but only on a simple memory of when resources were found or not. An individual moving according to dACS tends to move deeper into resource clusters and collect resources more efficiently than when utilizing the standard ACS. The later typically results in harvesting resources at the cluster's edge and often leads individuals to soon drift away from the resource cluster. The dACS can be interpreted as a survey movement within a resource cluster before switching from ESM to ISM. In addition, our results show that the duration of the transition from ISM back to ESM (half‐saturation constant) as well as resource density and cluster size, significantly influence foraging success and the optimal value of delay‐parameter. An individual with the added ability to estimate cluster size and resource density should perform even better than one applying a fixed‐rule dACS because it could optimally adjust delayed‐response movement steps and the duration of transition back to ESM. We believe that a delayed‐response ACS can occur in a wide range of taxa from organisms without a central nervous system (simple dACS) like protists to animals with complex brain systems including humans (adaptive dACS).

## Author Contributions

**Thotsapol Chaianunporn:** conceptualization (equal), data curation (lead), formal analysis (equal), funding acquisition (equal), investigation (lead), methodology (equal), resources (lead), software (lead), validation (equal), visualization (lead), writing – original draft (lead), writing – review and editing (equal). **Thomas Hovestadt:** conceptualization (equal), formal analysis (equal), funding acquisition (equal), methodology (equal), supervision (lead), validation (equal), writing – review and editing (equal).

## Consent

The authors have nothing to report.

## Conflicts of Interest

The authors declare no conflicts of interest.

## Data Availability

The data and the code of the simulation model that support the findings of this study are openly available in “Dryad” at https://doi.org/10.5061/dryad.vx0k6dk2r.
